# The Emergence of a vv + MDV Can Break through the Protections Provided by the Current Vaccines

**DOI:** 10.3390/v12091048

**Published:** 2020-09-20

**Authors:** Meng-ya Shi, Min Li, Wei-wei Wang, Qiao-mu Deng, Qiu-hong Li, Yan-li Gao, Pei-kun Wang, Teng Huang, Ping Wei

**Affiliations:** 1 Institute for Poultry Science and Health, Guangxi University, Nanning 530004, China; mengyashi12@126.com (M.-y.S.); liminmingd@163.com (M.L.); wangweiweihn@163.com (W.-w.W.); 1918402009@st.gxu.edu.cn (Q.-m.D.); 13879542109@163.com (Q.-h.L.); gaoyanli3@126.com (Y.-l.G.); tenhhwang@163.com (T.H.); 2 Institute of Microbe and Host Health, Linyi University, Linyi 276005, China; wangpeikun2018@163.com

**Keywords:** Marek’s disease virus, vv + MDV, pathogenicity analysis, immunosuppression, protection index

## Abstract

Marek’s disease (MD) is an infectious malignant T-cell lymphoma proliferative disease caused by Marek’s disease virus (MDV). In recent years, the emergence of very virulent (vv) and/or very virulent plus (vv +) strains of MDV in the field has been suggested as one of the causes of vaccination failure. The pathogenicity of the MDV strain GX18NNM4, isolated from a clinical outbreak in a broiler breeder flock that was vaccinated with CVI988/Rispens, was investigated. In the vaccination-challenge test, GX18NNM4 was able to break through the protections provided by the vaccines CVI988 and 814. It also significantly reduced body weight gain and caused marked gross lesions and a large area of infiltration of neoplastic lymphocyte cells in the heart, liver, pancreas, etc. of the infected birds. In addition, the expressions of programmed death 1 (PD-1) and its ligand, programmed death ligand 1 (PD-L1), in the spleens and cecal tonsils (CTs) of the unvaccinated challenged birds were significantly increased compared to those in the vaccinated challenged birds, indicating that the PD-1/PD-L1 pathway is related to immune evasion mechanisms. The results showed that the GX18NNM4 strain could cause severe immunosuppression and significantly decrease the protections provided by the current commercial vaccines, thus showing GX18NNM4 to be a vv + MDV strain.

## 1. Introduction

Marek’s disease (MD) is a lymphoproliferative disease of chickens caused by the highly infectious cell-associated alpha-herpesvirus, classified as Marek’s disease virus (MDV) [1]. MDV strains are classified into three species: *Gallid* herpesvirus 2 (MDV-1), *Gallid* herpesvirus 3 (MDV-2) and *Meleagrid* herpesvirus 1 (MDV-3) [1]. Among the three species, only MDV-1 is pathogenic and causes tumors in susceptible chickens. Since MD was first reported in 1907 [2], MDV has spread rapidly around the world, causing clinical disease, increased mortality and reduced growth, as well as sub-clinical immunosuppression, decreasing antibody levels of vaccinations [3,4]. MDV is an economically important disease, and it results in an estimated increased cost to the worldwide poultry industry of $1–2 billion annually [1,5]. However, MD has been well controlled by the use of MDV attenuated vaccines. These include the antigenically related herpesvirus of turkeys (HVT), MDV-2 strain SB-1 as well as the widely used MDV-1 strains CVI988/Rispens and 814 [6,7]. At present, CVI988 and 814 are considered the best vaccines in China [8,9]. The extensive use of MD vaccines has achieved significant success and reduced economic losses in the poultry industry [10,11].

A standardized MDV-1 pathotyping scheme based on the protection index (PI) provided by the HVT and HVT + SB-1 vaccine was developed at the US Department of Agriculture Avian Disease and Oncology Laboratory (USDA-ADOL) and later adapted to enable easier international comparison of isolates. MDV-1 strains are classified into four pathotypes, including mild (mMDV), virulent (vMDV), very virulent (vvMDV) and very virulent plus (vv + MDV) [12,13]. Some additional pathological characteristics include severe lytic infection, severe proliferative lesions, hemolytic anemia and tropism for monocytes. The pathogenesis of MD can be sequentially divided into four phases in vivo. During the early cytolytic phase, an initial lytic infection develops in B cells within 2–7 days post-challenge (dpc) [14]. MDV enters the latency phase in the CD4+ and CD8+ T cells where most of the viral gene expression is shut off between 7 and 14 dpc. At about 14–21 dpc, MDV reactivation in CD4+ T cells initiates a late cytolytic and immunosuppressive phase [15]. The transformation period around 28 dpc is characterized by the formation of visceral tumors that originate from CD4+ T-cell lymphomas [16]. MD isolates that differ in ability to induce MD lesions in vaccinated chickens also tend to differ in additional biological characteristics. Compared with mMDV, the more virulent isolates (v, vv or vv + MDV ) are generally considered to produce more visceral tumors with an earlier onset of tumor mortality, to cause more immunodepression often associated with severe lytic changes in lymphoid organs, to induce early non-neoplastic death and to replicate more rapidly and to higher titers [13]. The outcome of MDV infection in the host depends on a number of virus-determined and host-determined factors, and the pathotype of the virus is undoubtedly one of the most important factors. Recently, an inhibitory immunoreceptor, programmed death 1 (PD-1), and its ligand, programmed death ligand 1 (PD-L1), have been reported as molecules involved in immune evasion mechanisms for MDV pathogenesis [17]. PD-1 is expressed on activated T cells, B cells, NKT cells and myeloid cells [18]. PD-L1 is broadly expressed on both professional and non-professional antigen-presenting cells (APCs), and on lymphoid and non-lymphoid tissues [19]. The activation of these receptors is known to cause immune cell “exhaustion,” preventing the immune system from destroying infected cells [20]. 

In recent years, the emergence of vv and/or vv + MDV strains in the field has been suggested as one of the main causes of vaccination failure, which has caused new threats and greater losses to the poultry industry [21,22]. Guangxi is a major province in the production of high-quality chickens in China and has a significant resource of indigenous chicken breeds, called Yellow chickens, that are usually slow-growing, with long feeding periods, which can easily result in a higher risk of MD outbreak. According to the epidemiological data obtained in our group from 2017 to 2019, the isolation of MDVs increased dramatically in Yellow chicken flocks that had been vaccinated with CVI988 or 814 in southern China. However, the pathogenicity of such MDV epidemic strains is still unclear. In this study, we investigated the pathogenicity of MDV strain GX18NNM4, isolated from a breeder flock of Yellow chickens in southern China that had been vaccinated with CVI988/Rispens at hatching and had experienced depression, weakness, reduction in weight gain and an increased death rate after 120 days of age. The morbidity and mortality were 20% and 10%, respectively, at 140 days of age when this infection was diagnosed.

## 2. Materials and Methods

### 2.1. Virus and Vaccines

The GX18NNM4 (GenBank ID: MN943298) strain used in the present study was obtained from the peripheral blood lymphocytes (PBLs) that were separated from the anticoagulation-treated (adding sodium citrate) blood sample from the diseased bird [23]. GX18NNM4 was purified by plaque assay on a chicken embryo fibroblast (CEF) monolayer, and the *meq* gene sequence was also verified to eliminate the possible contamination of any vaccine virus as described previously [24,25]. These GX18NNM4-infected CEFs (at the fifth passage) were used for the challenge. They were characterized and identified as containing no contamination of avian leukosis virus (ALV), reticuloendotheliosis virus (REV) or chicken infectious anemia virus (CIAV), verified by PCR, immunofluorescent assay (IFA) and gene sequencing according to our laboratory routine [21,26]. The MD vaccines CVI988/Rispens and 814 were purchased commercially.

### 2.2. Experimental Design

A total of 175 day-old commercial Yellow chicken chicks without any prior vaccination were purchased from a local farm that was accredited by authorities for the elimination of specific avian diseases including avian leukosis (AL). The chicks were randomly divided into four groups, then individually housed in separate isolators and given free access to water and feed. The chicks in group I (*n* = 40) and group II (*n* = 40) were vaccinated with vaccines CVI988/Rispens and 814, respectively, on day one according to the manufacturer’s protocol. At seven days of age, the chicks in groups I, II and III (*n* = 58) were challenged intraperitoneally each with 200 μL of diluent containing 1000 plaque-forming units (PFUs) of the GX18NNM4 strain, while the chicks in group IV (*n* = 37) were inoculated with the same volume of Dulbecco’s minimum essential medium (DMEM) as the non-vaccinated and non-challenged control (Table 1).

### 2.3. Sample Collection and Processing

The birds were observed daily for the development of clinical signs, and all birds that died during the experiment or were sacrificed at the fixed time points of the experiment were examined postmortem. The MD-positive birds were estimated as previously described [27]. To determine the effect of MDV infection, the weights of the body and the PBLs, spleen, thymus and bursa were collected from three birds randomly selected from each group at 7, 14, 21, 28, 56, 72, 90 and 120 dpc. The relative weight of the immune organs (RWIO) was calculated using the following formula: RWIO = immune organ weight (g)/body weight (kg). Tumor-like tissues from various internal organs were fixed in 4% neutral buffered formalin solution, routinely processed and embedded in paraffin and used for the histopathological observation [28]. The total RNA was extracted from samples of the spleen and cecal tonsils (CTs) to detect the mRNA expression levels of PD-1 and PD-L1 [20,29,30]. 

### 2.4. Evaluation of Viral Loads in GX18NNM4-Challenged Birds by Real-Time PCR

The viral load of GX18NNM4 in the PBLs was quantified using a TaqMan probe real-time PCR assay that can distinguish vaccine strains, as described in Table 2. Total cellular DNA was extracted from the PBLs using the DNA extraction kit (TIANGEN, Beijing, China) according to the manufacturer’s protocol. Real-time PCR assays were performed using a FastStart Essential DNA Probes Master (Roche Diagnostics, Mannheim, Germany) and a LightCycler96 (Roche Diagnostics, Mannheim, Germany). 

### 2.5. Expression Analyses of the PD-1 and PD-L1 mRNA by Real-Time RT-PCR

The spleens and CT tissues of three birds of each group were homogenized in phosphate-buffered saline (PBS) (1:3 w/v), and then freeze-thawed three times at -80 °C/room temperature. Total RNA from the homogenized tissues was extracted using an AxyPrep™ Multisource Total RNA Miniprep Kit (Axygen Scientific Inc, Silicon Valley, USA), according to the manufacturer’s protocol. Each sample was treated with +gDNA wiper (Vazyme, Nanjing, China) to determine the PD-1 and PD-L1 expression levels of related factors by real-time RT-PCR. A master-mix consisting of 10 μL of hamQ Universal SYBR qPCR Master Mix (Vazyme, Nanjing, China) and 1 μL of cDNA was mixed with 10 pmol of each primer, 1 μL of *pp*38 probe and 6 μL of sterile water. The sequences of specific primers and accession numbers for PD-1, PD-L1 and the chicken *β-actin* are listed in Table 2 [31]. The amplification program was as follows: 95 °C for 180 s, 45 cycles at 95 °C for 10 s, 60 °C for 30 s, followed by 95 °C 4.4 °C /s 10 s, 65 °C 2.2 °C /s 60 s. Each sample was tested in duplicate, and the data are presented as averages. Relative quantification of the genes was determined using the 2^−ΔΔCT^ method [32]. 

### 2.6. Statistical Analysis

The birds with gross tumor lesions in the group of challenged birds without vaccination were counted. The vaccine’s PI was calculated by the following equation: PI = ((%MD in unvaccinated chickens – %MD in vaccinated chickens)/(%MD in unvaccinated chickens))×100, where %MD is the percentage of birds “at risk” of having MD lesions [12]. The absolute numbers of the MDV genome per million cells from the collected PBLs were normalized using the following formula: normalized MDV viral load = log10 ((MDV genome copy number/chicken genome copy number)×106) [27]. The data of body weight, along with the RWIO, the viral load and the mRNA expression of cytokines PD-1 and PD-L1, were also processed using GraphPad Prism (Version 8.0.1) and expressed as mean ± SD. One-way ANOVA was used to assess the differences among groups.

### 2.7. Ethical Statement

All procedures of animal experiments were approved by the Animal Welfare and the Animal Experimental Ethical Committee of Guangxi University (Approval No: GXU2019–081).

## 3. Results

### 3.1. Birds Infected with GX18NNM4 Show Significantly Inhibited Body Weight Gain and Severe Damage to the Immune Organs

**Figure 1:**
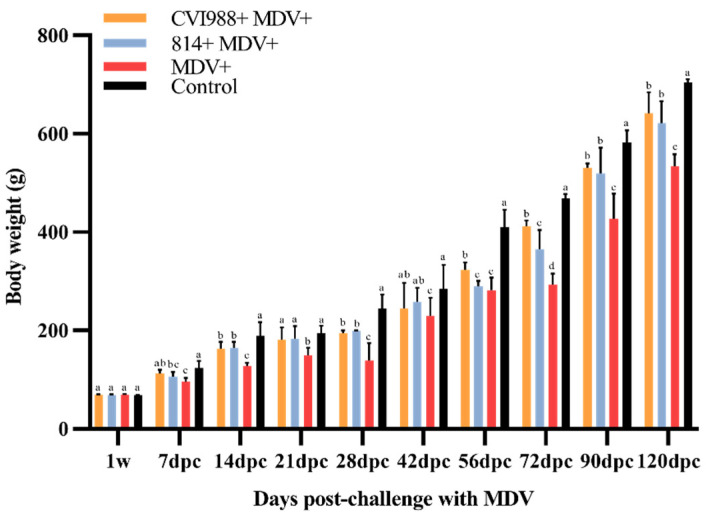
Statistics of body weight of experimental birds (X ± SD). Means not sharing a common letter are significantly different (*p* < 0.05).

**Figure 2:**
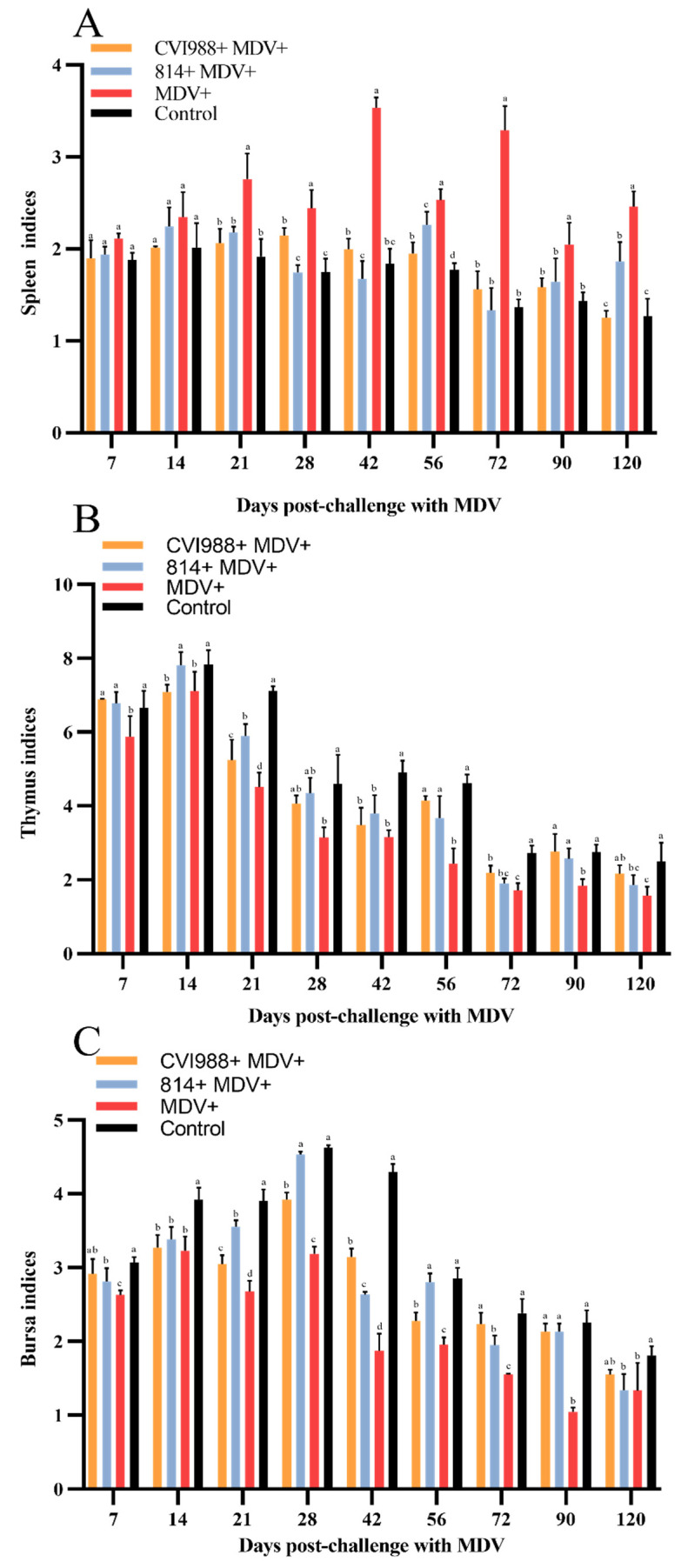
Dynamics of immune organ indices of the experimental birds. (**A**) spleen; (**B**) thymus; (**C**) bursa. Means not sharing a common letter are significantly different (*p* < 0.05).

As shown in Figure 1, the body weight (BW) of the birds in each group increased with time. The average BW of the birds in group I and II was lower than that of group IV at 14 dpc (*p* < 0.05); at 7 dpc and afterward, the BW of group III birds was significantly less than those of other groups (*p* < 0.05). The immune organs affected by MDV infection showed different degrees of injuries. The spleen of the infected birds in group III showed swelling, and the mean RWIO was significantly higher than those of other groups at 21 dpc and afterward (*p* < 0.05) (Figure 2A). The mean RWIO of the bursa and thymus in the infected birds of group III was significantly lower than those of other groups at 7 dpc (*p* < 0.05) (Figure 2B,C). The results showed that the birds challenged with GX18NNM4 exhibited serious damage to the immune organs.

### 3.2. The Viral Load in PBLs Was the Highest in Unvaccinated Challenged Birds

**Figure 3:**
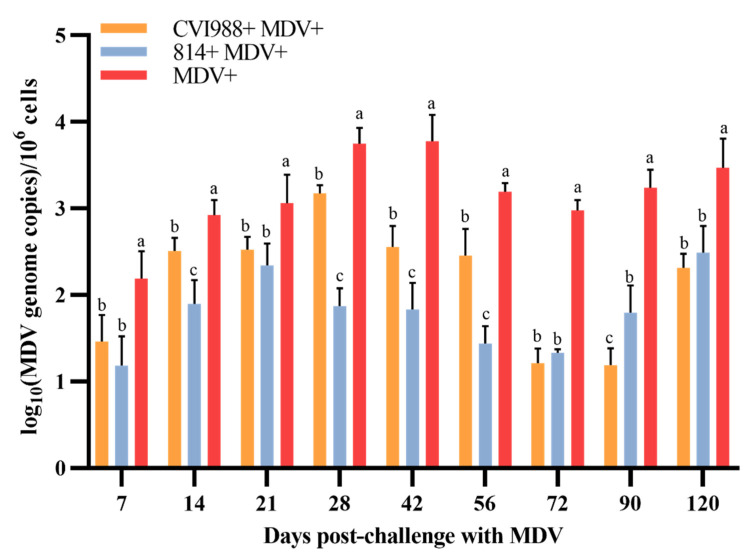
The viral load of MDV in the peripheral blood lymphocytes (PBLs) of the experimental birds. Means not sharing a common letter are significantly different (*p* < 0.05).

The viral load in PBLs of MDV in the infected groups rose between 7 and 28 dpc, and then tended to drop. During the whole experiment, the viral load of group III was higher than those of other groups at each time point and reached a peak at 28 dpc (*p* < 0.05) (Figure 3). The viral load of group I reached its peak at 28 dpc, and group II reached its peak at 21 dpc. Interestingly, the viral loads in the birds in group I were generally higher during the whole experiment than those in group II.

### 3.3. The Expression of PD-1 and PD-L1 Was Significantly Increased in the Unvaccinated Challenged Birds

**Figure 4:**
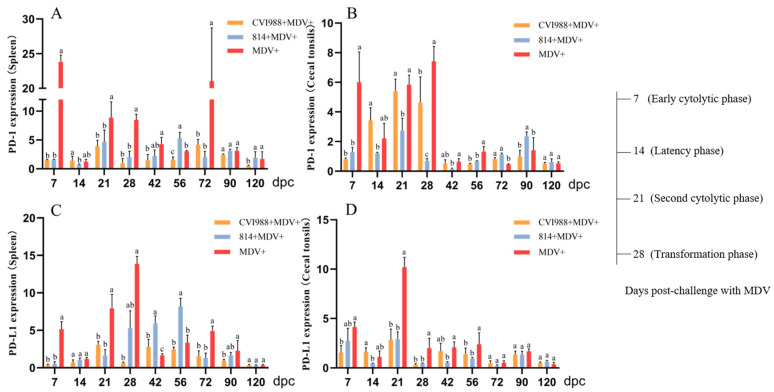
The mRNA expressions of PD-1 and PD-L1 in the tissues from birds experimentally infected with MDV. (**A**) PD-1 in the spleen; (**B**) PD-1 in the cecal tonsils; (**C**) PD-L1 in the spleen; (**D**) PD-L1 in the cecal tonsils. Determined by real-time PCR. Means not sharing a common letter are significantly different (*p* < 0.05).

The expression levels of PD-1 and PD-L1 in the spleens and CTs of the GX18NNM4-infected birds are shown in Figure 4. The expressions of PD-1 and PD-L1 mRNA increased in the early cytolytic phase (7 dpc) and decreased in the latency phase (14 dpc), and then upregulated again in the second cytolytic infection period (21 dpc) and the transformation period (28 dpc). Similarly, we observed a significantly lower expression of PD-1 and PD-L1 mRNA in the CTs than in the spleen, but the general trend was basically the same. Noticeably, the expressions of PD-1 and PD-L1 in the spleens and cecal tonsils of the unvaccinated challenged birds were significantly increased compared to those in the vaccinated challenged birds (*p* < 0.05).

### 3.4. The Gross Lesions and Histopathological Observations of the Experimentally Infected Birds

**Figure 5:**
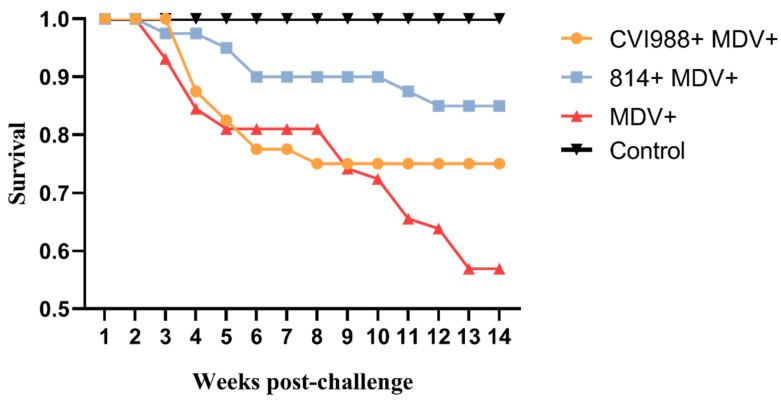
Survival patterns showing the overall effects of the various treatments.

**Figure 6:**
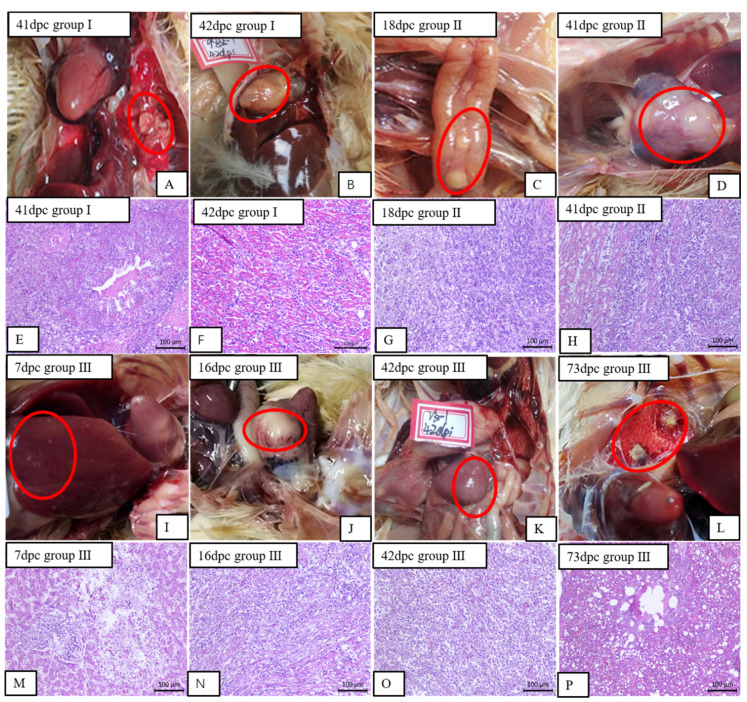
Anatomical and histological lesions: (**A**,**L**) white solid nodules in the lungs; (**E**,**P**) numerous proliferating tumor cells concentrated in the lung tissue; (**B**,**D**,**J**) tumor or tumor-like lesions in the heart; (**F**,**H**,**N**) numerous infiltrations of diffuse lymphocytosis in myocardial fibers; (**C**) pancreas hemorrhage with tumor; (**G**) numerous lymphocytosis proliferations in pancreas; (**I**) white tumor nodules, approximately 1–3 mm in diameter, on the liver surface; (**M**) numerous proliferating tumor cells were concentrated in the hepatic tissue; (**K**) white tumor nodule on the surface of spleen; (**O**) large number of lymphocytes infiltrated in the spleen tissue.

During the entire experimental period, the tumor tissues were collected from infected birds and examined histologically. Of the birds in all the groups, the earliest death was found in group III at 16 dpc, while the earliest deaths in groups I and II were at 22 and 18 dpc, respectively. The survival time in group III was generally shorter than those of groups I and II (Figure 5). All of the chickens were examined postmortem for the presence of tumors in the visceral organs. The results, which are presented in Table 3, showed that the majority of the birds had visible tumors in various visceral organs. These tumors were most easily observed in the heart and pancreas, followed by the liver, caecum, spleen and lung. The internal organs were observed to have gross lesions and large areas of infiltration of neoplastic lymphocyte cells in all the infected birds (Figure 6). 

### 3.5. Birds Infected with GX18NNM4 Can Exhibit Severe Tumor Incidence and Break through the Protections Provided by the Current Vaccines

A high incidence of tumors was found in all the birds infected with GX18NNM4. As shown in Table 3, no death or tumor was found in group IV during the experiment. The GX18NNM4 strain induced tumor development in 70.7% of the chickens in group III. These tumors were most commonly found in the heart (34.5%) and liver (32.8%), followed by the pancreas (27.6%), spleen (17.2%), caecum (17.2%) and lung (13.8%). The morbidity (27.5%) and tumor incidence rate (27.5%) of group II were significantly lower than those of group I (45% and 42.5%, respectively, *p* < 0.05), indicating that the PI of vaccine 814 (61.1%) was higher than that of vaccine CVI988 (39.9%) (*p* < 0.05), though neither of the vaccines could provide full protection against the challenge of the GX18NNM4 strain. 

## 4. Discussion

MD is a severe neoplastic disease caused by MDV that adversely affects the poultry industry. In the past decade, it has been reported that there are many clinical tumor cases caused by highly virulent MDV strains in MD-vaccinated chicken flocks [21,27,33,34,35]. In addition to certain improper operations and the infections of other immunosuppressive disease viruses, the emergence of highly virulent strains was suggested as the main cause of the failure of MD vaccination, which brought more and more attention to the pathogenicity of MDV wild strains [21,27,35]. It is worth noting that the Guangxi province is a major source of production of Yellow chickens in southern China, and about 9 billion birds were produced in 2019, an increase of 16.7% from 2018. This is significant as chicken production is one of the main alternatives to pork, which has had reduced production due to the outbreak of African swine fever [36]. In this study, the MDV strain GX18NNM4 was isolated from a CVI988-vaccinated broiler breeder flock, which experienced severe MD. In order to further understand the virulence of the newly epidemic strains of MDV in Guangxi, the pathogenicity of the newly isolated MDV strain GX18NNM4 was investigated.

In our study, the GX18NNM4 strain was found to significantly inhibit body weight gain (*p* < 0.05) and to cause severe damage to the immune organs (thymus, spleen and bursa), resulting in severe immunosuppression. The expressions of PD-1 and PD-L1 mRNA were increased in the early cytolytic phase, but dramatically decreased following the onset of the latent infection. In humans, PD-1 is found on the surface of T cells. Healthy cells have a PD-L1 which, when bound to PD-1, inactivates the immune cell, thus stopping it from destroying healthy tissue. However, some viruses can increase the number of PD-L1 receptors displayed by infected cells, thereby preventing their destruction and allowing the virus to continue to replicate. Combined with previous studies [31], we found that the expressions of PD-1 and PD-L1 were significantly increased in the unvaccinated challenged birds, allowing the virus to evade the immune mechanism of the host and continue to replicate, indicating that this might be one of the pathogenic mechanisms of the GX18NNM4 strain. 

The GX18NNM4 strain can break through the protections provided by the current commercial vaccines with high morbidity and mortality. In brief, the morbidity, the degree of MD lesions and damage to immune organs and the viral load in the 814 vaccinated group were relatively less than those in the CVI988 vaccinated group. The survival rate and PI in the 814 vaccinated group were higher than those in the CVI988 vaccinated group. The 814 vaccine seems to have better protection against this Guangxi isolate in Yellow chickens. According to previous research in our laboratory, based on the analysis of the phylogenetic tree and SNP of several antigenic gene sequences, the Guangxi MDV field isolates have a closer relationship to the Chinese vaccine strain 814 than to the CVI988 strain [34]. According to the definition of virulence [12,37], we designate the isolate GX18NNM4 as belonging to the vv + MDV category. One of a series of studies since 2008, this study used the newly isolated local strain GX18NNM4 to conduct the pathogenicity studies on Yellow chickens. In contrast to the other previous vv/vv + MDV strains GXY2, YL040920 and LTS, the GX18NNM4 strain could cause acute death with marked gross lesions in the pancreas and lung, and the vaccines’ protections were even less effective [22,38,39]. With more and more vv + MDV strains being isolated from vaccinated chicken flocks and the complex infections arising from the combination with ALV and/or REV pathogens, more serious diseases and losses in flocks have been found, which has brought new threats and greater economic loss to the poultry industry [21,40].

In conclusion, the emerging MDV epidemic strain GX18NNM4 (vv +), isolated from Yellow chickens, was found to cause severe tumor incidence and to break through the protections provided by the current commercial vaccines. We also revealed that the PD-1/PD-L1 pathway might be involved in the immunoinhibitory functions of vv + MDV infection. There is a need to develop new vaccines and to reinforce strict biosecurity against vv/vv + MDV in the field, especially for the Yellow chickens, in order to prevent and control the recent outbreaks of the disease.

## Figures and Tables

**Table 1:** The vaccinations and challenges of the experimental groups.

Group	No. of Birds	Vaccine (Day 1)	MDV Challenge (Day 7)	PFU per Volume (μL)
I	40	CVI988/Rispens	GX18NNM4	1000 per 200
II	40	814	GX18NNM4	1000 per 200
III	58	DMEM	GX18NNM4	1000 per 200
IV	37	DMEM	DMEM	0 per 200

PFU: plaque-forming units.

**Table 2:** Primers used for the real-time PCR.

Gene	Type	Sequence (5′-3′)	Accession Number	Reference
*pp38*	Forward	ATAAAGGGTGATGGGAAGGC		Our group’s designs (unpublished)
	Reverse	CGTCAAGATGTTCATTCCCTG		
	Probe	TCCTCCCACTGTGACAGCC		
*PD-1*	Forward	GGACTACGGTGTGCTGGAGTT	XM422723	Matsuyama-Kato et al., 2012
	Reverse	TCTTTCCTCGCTCTGGTGTG		
*PD-L1*	Forward	TTCAGGGACGGATAAAGCTG	XM424811	
	Reverse	CGTCTCTGAGCTTCACGTTG		
*β-actin*	Forward	GAGAAATTGTGCGTGACATCA	NM205518	Our group’s designs (unpublished)
	Reverse	CCTGAACCTCTCATTGCCA		

**Table 3:** The disease incidences of the birds challenged with the GX18NNM4 strain.

Group	Heart	Liver	Spleen	Lung	Pancreas	Caecum	Morbidity	Mortality	Tumor Rate	PI
I	22.5% (9/40)	12.5% (5/40)	7.5% (3/40)	5% (2/40)	20% (8/40)	17.5% (7/40)	45% (18/40)	25% (10/40)	42.5% (17/40)	39.9%
II	15% (6/40)	7.5% (3/40)	5% (2/40)	0	7.5% (3/40)	10% (4/40)	27.5% (11/40)	15% (6/40)	27.5% (11/40)	61.1%
III	34.5% (20/58)	32.8% (19/58)	17.2% (10/58)	13.8% (8/58)	27.6% (16/58)	17.2% (10/58)	75.9% (44/58)	43.1% (25/58)	70.7% (41/58)	--
IV	0	0	0	0	0	0	0	0	0	--

PI: protection index.
